# Improving access to psychological therapies and older people: Findings from the Eastern Region

**DOI:** 10.1016/j.brat.2014.03.008

**Published:** 2014-05

**Authors:** A. Matthew Prina, Riccardo E. Marioni, Geoffrey C. Hammond, Peter B. Jones, Carol Brayne, Tom Dening

**Affiliations:** a Department of Public Health and Primary Care, Institute of Public Health, University of Cambridge, Robinson Way, University Forvie Site, Cambridge CB2 0SR, UK; b NIHR Collaboration for Leadership in Applied Health Research and Care for Cambridgeshire and Peterborough (CLAHRC-CP), UK; c Department of Psychiatry, University of Cambridge, UK; d Division of Psychiatry, Institute of Mental Health, University of Nottingham, UK; e King's College London, Institute of Psychiatry, Health Service and Population Research Department, Centre for Global Mental Health, London SE5 8AF, UK

**Keywords:** Anxiety, Depression old age psychiatry, General practice, Accessibility, CBT, Waiting times, Psychological therapies

## Abstract

**Background:**

Evaluations of the Improving Access to Psychological Therapies (IAPT) scheme have not yet focused on minority subgroups. This paper aims to evaluate accessibility, waiting times and clinical outcomes of IAPT for older adults.

**Methods:**

All referrals from six Primary Care Trusts (PCT) in the East of England were used in this analysis. During each session, the therapist recorded information on anxiety symptoms using the Generalised Anxiety Disorder Questionnaire (GAD-7) and depressive symptoms with the Patient Health Questionnaire (PHQ-9). Waiting times, type of referrals and reliable recovery rates were investigated.

**Results:**

Older adults accounted for only 4% of all the IAPT referrals made between September 2008 and July 2010 in the Eastern Region. Waiting times for both IAPT assessment and treatment were slightly lower for older adult. In all centres, reliable recovery rates were higher in older adults compared to younger adults post-treatment, however these differences were not significant, with the exception of a difference in anxiety scores (*χ*^2^(1) = 18.6, *p* < 0.001). In multivariate analyses, being an older adult was associated with recovery for depression (OR = 1.30, 95% CI 1.10–1.53), anxiety (OR = 1.42, 95% CI 1.21–1.66), and overall recovery (OR = 1.31, 95% CI 1.10–1.54) after adjustment for gender, PCT region, baseline score, maximum treatment step during treatment, dropping out, and number of sessions.

**Conclusions:**

The IAPT services were shown to be beneficial to older patients, however, access to these services in later life has been lower than expected. The service pathway for older populations needs to be better researched in order to eliminate possible obstacles in accessing services.

## Introduction

Anxiety and depression are two highly prevalent mental conditions in adults. Both conditions have been shown to be leading contributors to disability (Bijl & Ravelli, 2000; Prina, Ferri, Guerra, Brayne, & Prince, 2011a), are associated with an increased risk of other physical illnesses (Knol et al., 2006; Wulsin & Singal, 2003), and have a major impact on quality of life (Beard, Weisberg, & Keller, 2010; Rapaport, Clary, Fayyad, & Endicott, 2005). Anxiety and depression remain common throughout the lifetime with estimates among older people varying from 13.5% for depression (Beekman, Copeland, & Prince, 1999) to 1–15% for anxiety disorders (Bryant, Jackson, & Ames, 2008; Prina, Ferri, Guerra, Brayne, & Prince, 2011b). The type of treatment offered for common mental disorders is similar for both younger and older adults, with pharmacological and psychological interventions being the most common. Psychological interventions have been used successfully for the treatment of anxiety disorders in older age, as demonstrated by the large number of meta-analyses that have shown the benefits of interventions over control conditions (Goncalves & Byrne, 2012; Hendriks, Oude Voshaar, Keijsers, Hoogduin, & van Balkom, 2008). Psychological therapies have also been shown to be effective in the treatment of depression in later life (Krishna et al., 2011; Pinquart, Duberstein, & Lyness, 2007). A recent report highlighted that psychological therapies are equally effective in the treatment of depression in younger and older adults (Cuijpers, van Straten, Smit, & Andersson, 2009). This may not be the same for anxiety, with a meta-analysis of Cognitive Behavioural Therapy (CBT) showing a lower efficacy in older versus working-age adults (Gould, Coulson, & Howard, 2012). However, the disparity in findings may have been driven by methodological differences (Gould et al., 2012); these treatments may be equally effective across the life spectrum.

Partly based on the effectiveness of psychological therapies for the treatment of common mental disorders, a large-scale scheme for Improving Access to Psychological Therapies (IAPT) for people suffering with mild or moderate anxiety and depression was announced within the English National Health Service in October 2007 and piloted in Doncaster and Newham in Greater London. The IAPT service is based on a stepped-care model, as recommended by the National Institute for Health and Clinical Excellence (NICE) (NICE, 2011). Step 2 (“treatment of mild depression in primary care”) and Step 3 (“treatment of moderate to severe depression in primary care”) are the main focus of the IAPT services. Low intensity interventions are delivered during Step 2 by a mix of workers with a wide range of backgrounds who have trained as Psychological Wellbeing Practitioners (PWPs). The types of therapies available in Step 2 can be delivered by either face-to-face contact or telephone support and include bibliotherapy, behavioural activation, guided cognitive behavioural self-help, guided self-directed exposure therapy, and computerised CBT. Step 3 is used mostly for moderate to severe depression and is generally delivered by CBT competent professionals. Individual CBT, group CBT, therapy sessions with guided self-help and medication advice can all be during this step.

A specific aim of the IAPT programme was to relieve the stress and financial costs associated with mood disorders. These were estimated at approximately £ 150 billion alone for depression in 2009/2010 (The Centre for Economic Performance's Mental Health Policy Group, 2006) across the country. The IAPT programme is based on the concept that an improvement in mental health across the UK would result in economic gains related to increased productivity and re-employment of those individuals unable to work due to mental illnesses (Clark et al., 2009). Layard (2006) claimed “the cost to the government would be fully covered by the savings in incapacity benefits and extra taxes that result from more people being able to work”. The cost-benefit assumption that was made at the inception of IAPT is probably more applicable to working-age adults rather than to older adults, a group of people unlikely to re-enter the work market. However, under the Equality Act 2010, public bodies are not allowed to discriminate access to services on the basis of age. IAPT services are no exception.

The economic argument however may also be valid for older adults. Indirect cost savings related to hospitals and carers could be used to argue for better recognition and treatment of common mental disorders in older age. A recent paper has shown that older adults with depression have much higher hospital care costs than people without depression (Prina et al., 2014). Moreover, many older adults are also involved in other ways besides paid employment, for example in voluntary roles or in supporting family members (e.g. with child care, enabling parents to work) (Royal Voluntary Service (WRVS), 2011). It is therefore important that older adults are able to access services, not only on moral grounds, but also on quality of life grounds and potential cost savings to health services, and more broadly to society.

In this paper, we aim to explore possible differences in referrals and waiting times to access IAPT services between younger and older adults, using data from Primary Care Trusts (PCTs) in the East of England. Clinical outcomes and differences in clinical recoveries are also explored.

## Methods

### Data extraction

The study aimed to include all the referrals to IAPT services in the East of England between September 2008 and July 2010. Five of the 12 Primary Care Trusts (PCTs) had been commissioning the service for less than 12 months, so data were used only from the remaining seven PCTs with stable services by September 2008. One further PCT was removed from the analyses as it did not include any adults over the age of 65. The remaining six PCTs were: Bedfordshire, Mid-Essex, North-East Essex, North-East Hertfordshire, Suffolk, and West Hertfordshire. Each PCT was anonymised and given a corresponding letter from A to F, using a similar approach as the one used in a previous paper (Hammond et al., 2012).

### Measurements

During each session, therapists uploaded information onto the Patient Case Management Information System (PC-MIS), the routine clinical data system used within IAPT. The information captured at each session contributed to the IAPT minimum data set. This included information on socio-demographics, attendance, source and date of referral, date of appointment, primary diagnosis and treatment outcomes.

Symptom severity was assessed using two different scales. Anxiety symptoms were measured using the Generalised Anxiety Disorder Scale (GAD-7). This is a seven-item scale, with each item scored from zero (“Not at all bothered by the problem”) to three (“Bothered nearly every day”). The cumulative score on this scale can range from 0 to 21. A score of eight or higher on this scale has been shown to correspond to a clinical diagnosis of anxiety (Spitzer, Kroenke, Williams, & Lowe, 2006).

The Patient Health Questionnaire Depression scale (PHQ-9), a nine-item scale, was used to assess symptoms of depression. The item scoring for this scale is similar to the GAD-7, with responses ranging from zero to three. The sum score can range from 0 to 27. A score of 10 has been used as a diagnostic threshold for clinical depression (Lowe, Kroenke, Herzog, & Grafe, 2004).

Information on socio-demographic data was also included in the IAPT minimum data set and included gender, age and ethnicity. Ethnicity grouped into White, Mixed, Asian, Black, and ‘Chinese or other’.

### Statistical analysis

The database used for our analyses included every adult referred to IAPT who had at least two scheduled sessions, and for whom the GAD-7 and the PHQ-9 scales had been completed.

For this study, we subdivided the sample in two age groups: those aged between 18 and 65, and those over 65. We compared and contrasted clinical indicator scores (PHQ-9 and GAD-7) and outcomes (waiting times, source of referrals, recovery).

Source of referrals and waiting times compared between the two groups using Chi-Square tests of independence. Both time to first assessment and time to first treatment were calculated using the referral date as time zero. Mean and median times were calculated with their respective standard deviations (SDs) and interquartile ranges (IRQs). Mann–Whitney *U* tests were used to compare median times between the two different populations. The sources of referrals were grouped in the following categories: general practitioners, other clinical specialties, health visitors, self-referrals, others.

Because the sampling criteria of two or more completed sessions may have distorted true dropout rates, we included all the individuals who had their referrals accepted, regardless of whether or not they had completed two sessions.

Recovery rates for both the PHQ-9 and GAD-7 were calculated separately for different PCT areas and age groups, using the reliable recovery index described by Gyani, Shafran, Layard, and Clark (2013). This index was created to avoid misclassifying individuals who were just above the cut-off point at the initial assessment as ‘recovered’ even though their improvement was not statistically reliable. An individual was considered to have recovered if the final score was below the cut-off point for ‘caseness’ in the PHQ-9 (10) or the GAD-7 (8) and there was a reliable improvement during treatment. The improvement was considered reliable if the change in scores between the initial and the last assessment was above 5.20 on the PHQ-9 and 3.53 on the GAD-7. ‘Overall recovery’ was defined as scoring below the clinical cut-offs on both the PHQ-9 and the GAD-7, and to have shown reliable improvement on the PHQ-9 or GAD-7, with the score in the other scale also declining or not reliable worsening. Chi-square (*χ*^2^) tests were used to compare recovery rates between the age populations. Finally, to investigate the role of confounding variables, we modelled potential factors associated with recovery using logistic regression. Age group, gender (male/female), PCT region, maximum number of sessions, baseline score, treatment step (2 or 3) and dropping out were all included in the final model. The treatment step corresponds to NICE steps 2 and 3 for the treatment as described in the previous section.

## Results

### Referrals

**Fig. 1:**
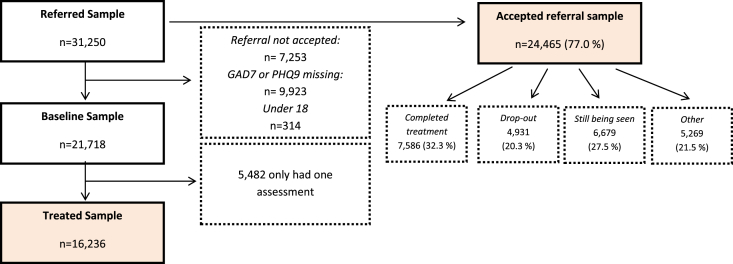
Number of excluded individuals from each sample.

Between September 2008 and July 2010, 31,574 individuals were referred to the IAPT programme in seven PCTs of the East of England region. Of these, 314 (0.9%) were aged less than 18, 30,023 (95.1%) were aged 18–65, and 1227 (3.9%) were aged over 65. Referrals were declined for 7253 individuals (23.0%) and no significant difference was observed in the percentage declined between the older (23.4% referrals declined) and the 18–65 sample (22.0%), (*χ*^2^(1) = 23.7, *p* > 0.05). Of all the people who had their referrals accepted (Fig. 1), only 32.3% completed their treatment as of July 2010, with 20.3% of individuals dropping out of treatment and 27.5% still being seen at the cut-off date of July 2010. 53.6% of older adults completed treatment, compared with only 36.3% of adults of working age (*χ*^2^(1) = 139.1, *p* < 0.01). This difference may have been due to variations in treatment dropout rates, with only 10.2% dropout in older adults compared to 24.6% in younger ones (*χ*^2^(1) = 88.8, *p* < 0.01).

Fig. 1 shows a flow diagram of exclusions for analyses assessing differences in symptom severity measures. The characteristics of the different populations have also been compared and highlighted in Appendix 1.The final ‘treated’ sample was 16,236. In this sample, women outnumbered men, and the majority of patients were of White ethnicity (Table 1). Asian and Asian British were the second largest ethnic group, followed by Black and other groups. Overall, mixed anxiety and depression (26.0%), major depressive disorder (21.8%) and generalised anxiety disorder (15.3%) were the top diagnoses. In around 35% of patients a diagnostic code was missing.

The majority of patients were referred to IAPT services from their General Practitioners (GPs), followed by self-referrals, referrals from other clinical specialties, and health visitors (Table 2). There were differences between older and younger adults with respect to source of referral. The percentage of older adults referred to the IAPT service by their GPs was 84.5%, compared with 87.4% of younger adults (*χ*^2^(1) = 5.0, *p* = 0.02). Self-referrals were higher among older adults (8.5%) compared with those aged 18–65 (6.2%) (*χ*^2^(1) = 5.3, *p* = 0.02) (Table 2).

### Expected rate of access in people aged over 65

In order to calculate the expected rate of access in people aged over 65, we estimated the differences in the age structure in the Eastern region based on 2011 census data. There were 446,000 people aged 65 or over (20%), compared to 1,731,200 (80%) adults aged 16–64. The most recent UK estimates of common mental disorders (CMDs) are from the Adult Psychiatric Morbidity Survey (McManus, Meltzer, Brugha, Bebbington, & Jenkins, 2009; Spiers et al., 2011), with a prevalence of CMDs of 10.3% in over-65s, compared with 17.2% in younger adults. Based on the data, we can assume that 2% of the sample is expected to be older and have a CMD, compared to 13.8% expected to be younger and have a CMD. Given the differences in the prevalence of CMDs and the age profile of the population in the Eastern Region, the expected rate of access to the IAPT service in older people should be 12.7%, compared to the 3.9% that was seen above.

### Waiting times

In order to assess waiting times, two definitions were used: time from referral to first assessment and time from referral to first treatment. Mean and median times to assessment were 46.5 days (SD = 53.8) and 28 days (IQR = 14–57), respectively. The mean time to treatment in the sample was 51.1 days (SD = 57.5), whereas the median was 33 days (IQR = 15–64). Mean and median times differed between older and younger adults, with older adults having to wait less time before receiving an assessment (*z* = 2.4, *p* = 0.02) or treatment (*z* = 3.1, *p* = 0.002) (Table 3). There were also differences in waiting times when the maximum step received during the course of the treatment was taken into account (Table 3). Experiencing a longer waiting time to either assessment or treatment was not associated with an increased likelihood of dropping out, with ORs of 1.00 (95% CI: 0.99–1.00) after adjustment for age, and baseline clinical scores.

### Clinical outcomes

Table 4 shows differences in recovery rates for anxiety and depressive symptoms, stratified by age and PCT. Recovery here is defined as being below the clinical cut-off for each scale, and showing reliable improvement during treatment. In all PCTs, recovery rates were higher in older adults compared to younger adults, however these differences in reliable recovery were not significant, with the exception of a difference in anxiety scores (*χ*^2^(1) = 18.6, *p* < 0.001). When recovery was assessed across both scales (i.e. one of the GAD-7 and PHQ score reliably decreased and the score for the other scale did the same or did not reliable deteriorate), 32.1% of older adults had a reliable recovery, compared to 29.1% of younger adults (*χ*^2^(1) = 2.91, *p* = 0.088). Recovery varied between different PCTs. Overall reliable recovery ranged from 33.3% for PCT D to 49.1% in PCT A (*χ*^2^(5) = 158.0, *p* < 0.001).

### Confounding factors

In order to investigate factors associated with recovery, multivariate logistic regression models were run. Being aged between 66 and 85 was associated with increased odds of recovery from anxiety (OR = 1.42, 95% CI 1.21–1.66), depression (OR = 1.30, 95% CI 1.10–1.53), and overall reliable recovery (OR = 1.31, 95% CI: 1.10–1.54), after adjustment for gender, PCT region, baseline score, maximum treatment step during treatment, number of sessions and dropping out (Table 5). Undertaking a higher number of sessions, having a higher initial baseline score and not dropping out were also associated with improved recovery (Table 5).

## Discussion

This study showed that older adults accounted for only 4% of all IAPT referrals made between September 2008 and July 2010 in the Eastern region. People aged over 65 were less likely to be referred to IAPT from their GPs, compared with adults of working age, and they were more likely to refer themselves. Dropouts from treatment and waiting times were also reported to be lower in this age group. Recovery rates varied across PCTs but they were generally better among older patients.

### Strengths and limitations

One of the major strengths of this study lies in its large sample size that included over 16,000 individuals and data from almost 100,000 sessions over a two-year period. Six PCTs were represented covering a major part of this region. The population covered is representative of the east of England, which altogether comprises almost 10% of the population of England. Although it is difficult to generalise the findings to other locations across the United Kingdom, fixing the site effects did not impact the likelihood of the recovery for older adults. This suggests that similar outcomes may be found in other areas outside this region.

The relatively high percentage of missing data in this sample is a major limitation of the study. Routinely collected data are often subject to incompleteness, and are very unlikely to reach the low percentages of missing data seen in some research studies. A large proportion of missing data were present for the ethnic categories. This hindered any further analysis based on this information. Completeness of this variable is important and should be achieved by the therapists who record the data during the assessment, if important questions relating to access and outcomes of ethnic minorities are to be addressed.

The largest limitation of this outcome analysis is intrinsic to the study design itself and relates to the use of symptom severity measures as proxies for diagnoses. Although these scales have been validated (Kroenke, Spitzer, Williams, Monahan, & Lowe, 2007) and have shown moderate specificity and sensitivity, they are not replacements for clinical diagnoses. The PHQ-9 in particular has been shown to have high specificity but low sensitivity, and it could therefore miss some patients with depression (Wittkampf, Naeije, Schene, Huyser, & van Weert, 2007). Another problem with short symptom rating scales is that they are not often able to incorporate the clinical spectrum of symptoms seen in older adults (Baldwin, 2008). The PHQ-9 scale was recently validated in an older population, showing similar specificity and sensitivity to the Geriatric Depression Scale (GDS) (Phelan et al., 2010). Interestingly, an optimum cut-off point of nine was found, compared with ten that was used in this study. This could explain why older adults were less likely to have lower baseline scores than younger adults. However, the lack of a nationally representative sample, a small sample size and a very low prevalence of depression, somewhat limit the generalisability of this validation (Phelan et al., 2010).

It is possible the improvements in symptoms may not be genuine effects. These changes could represent regression to the mean or natural resolution of symptoms. However, the efficacy of psychological interventions has been shown in randomised controlled trails, therefore suggesting that these changes could denote real treatment effects.

Finally, a large number of patients ended treatment after only one session, which certainly raises some questions as to why there are so many dropouts who do not complete treatment. The large number of dropouts could have also affected the generalisability of our analysis. Given the close proximity and the high number of sessions, a problem with test-retest bias, where scales are administered on multiple occasions in a short time period, could also arise.

### Interpretation of findings

A very small percentage of older subjects accessed the IAPT services in the Eastern region. We found that of all the people accessing the IAPT service, only 4% were older adults. We expected this proportion to be closer to 13%, based on the age structure of the Eastern region and the prevalence found in the Adult Psychiatric Morbidity Survey. This expected rate is however likely to be an underestimate, as the calculations are based on the assumptions that the prevalence of CMDs in these age groups is accurate. This survey only included households, and excluded hospitalised and institutionalised subjects, suggesting that the true-estimate of CMDs in over-65s may be higher.

Referrals made by general practitioners were lower for older people. This is probably due to the fact that GPs are generally less attuned to identifying mental health problems and needs in older patients. This has been clearly reported in the literature (Shah, McNiece, & Majeed, 2001; Watts et al., 2002). One of the potential sources of referrals in the IAPT programme is via self-referral. This was introduced to provide another route into services and to target individuals or minorities who would not otherwise access traditional services. In this regard, the results from this study are somewhat encouraging, showing that groups who would not traditionally be captured can be targeted by increasing the type and sources of referrals. One of the PCTs included in our evaluation did not allow for self-referrals, and it is likely that similar exclusions are present across the country. This needs to be addressed. A recent paper by Brown, Boardman, Whittinger, and Ashworth (2010) has highlighted the positives and negatives of a self-referral system in IAPT, concluding that this system is mostly advantageous, bolstering access to harder to reach communities, and to those who never thought of consulting a GP, because of stigma, pre-conceived attitudes towards doctors, or health beliefs.

Differences in waiting times for both treatment and assessment were also found between various age groups. The shorter waiting times for older adults could potentially be attributed to lower depression and anxiety scores at baseline, however this is unlikely to be the case. Unfortunately, we were not able to test this in the database. Waiting times were reduced not only for treatment but also for assessment, and differences were still seen when the maximum step of treatment, which relates to the severity of the condition, was taken into account. It is possible that older people may be more compliant with appointments offered and younger people may be harder to arrange a time with because of work and other commitments, or ambivalent motivation. Further research may be able to explain why this might be the case.

Finally, recovery rates for both anxiety and depression among older adults were shown to be higher than in younger adults, across most PCTs. The differences were not statistically significant in many cases, with the exception of a reliable recovery on the anxiety scale, but this is probably due to the small sample sizes in some of the sites. Age was however associated with recovery for depression, anxiety and overall recovery when introduced in a multivariate model. As expected, having a higher number of sessions, and receiving a lower treatment step were both associated with better recovery. Older adults were also shown to be less likely to dropout of treatment, and this could be a feasible suggestion as to why recovery rates are better in this group. Furthermore, dropping out of treatment was negatively associated with recovery. Systematic reviews have shown that psychological therapies are effective in the treatment of depression and anxiety in older adults (Cuijpers et al., 2009; Goncalves & Byrne, 2012; Hendriks et al., 2008; Krishna et al., 2011; Pinquart et al., 2007). The findings from this service evaluation seem to support these reviews, even though this is not an experimental study, and it is difficult to attribute any difference to the treatment alone. Our findings however do not seem to reflect those from Gould et al. (2012) that psychological therapy for anxiety may not be as effective in the older age group as in working-age. The suggestion that methodological issues and in particular the lack of adjustment for baseline score is behind the findings of this reviews, seems to be confirmed in our sample where, after adjustment for baseline score and other confounders, the odds ratios for recovery in the older age group increased of magnitude. This data may be consistent with evidence that older people are better at emotional regulation competences in comparison to working-age adults (Carstensen et al., 2011).

A small systematic review of behavioural therapy in older adults (Samad, Brealey, & Gilbody, 2011) recently showed that CBT seems to have comparable effectiveness with alternative psychotherapies in older age, even though only four studies were included. Further research investigating different forms of psychotherapies between different age groups are therefore warranted, and also the investigation of factors that limit CBT effectiveness in diverse groups.

### Conclusions and future research

The major aim of IAPT was to reduce waiting times and improve access to psychological therapies in its target population of working-age adults. In this initial evaluation, these services were shown to be beneficial to older patients. If further research confirms these findings, the economic argument that by improving mental health across the population the productivity is increased and consequently the economy of the country could be made for older adults too. The idea that older people do not contribute to the economy is too simplistic.

Access to the IAPT services for older adults is lower than expected, given household survey estimates of the prevalence of depression. It probably reflects how these services were set up but, given the outcome data presented above, this limited access needs attention in order to address age discrimination in service access.

Better awareness among GPs of psychological problems in later life, including dementia should be raised and be on future agendas. Development of other potential points of access to psychological therapies for people in institutions, or other facilities, also need increased exploration.

Future research will need to address what type of treatments work better for different groups of older adults in varying settings, as not everything offered by IAPT services may work well in older age. For example, self-help using existing materials might be problematic in older adults with visual impairments, but these are only small barriers that can be overcome if we want to remove the misperception that psychological therapies do not benefit older people (Bhutani, 2008, chap. 19).

## Figures and Tables

**Table 1:** Socio-demographics characteristics of the sample.

	18–65 *n* = 15,065	66–85 *n* = 705	*p* Value
Gender (female %)	9956 (66.1)	480 (68.0)	*p* = 0.73
*Missing*	277(1.8)	7 (1.2)	

PCT			
A	2159 (14.3)	116 (16.4)	*P* < 0.05
B	1649 (10.9)	86 (12.2)	
C	1964 (13.0)	64 (9.1)	
D	1685 (11.2)	102 (14.5)	
E	4688 (31.1)	211 (29.9)	
F	2920 (18.8)	126 (17.9)	
*Missing*	0	0	

*Ethnicity*			
White	11,169 (96.4)	524 (97.8)	*p* = 0.222
Mixed	126 (1.1)	1 (0.2)	
Asian	161 (1.4)	5 (0.9)	
Black	60 (0.5)	4 (0.7)	
Chinese or other ethnic group	66 (0.6)	2 (0.4)	
*Missing*	3483 (23.1)	169 (24.0)	

**Table 2:** Source of referrals for the treated sample.

	18–65	>65	*p* Value
GP	12,866 (87.2%)	577 (84.5%)	*p* > 0.05
Self-referral	944 (6.4%)	58 (8.5%)	
Other clinical specialty	525 (3.6%)	26 (3.8%)	
Health visitor	176 (1.2%)	11 (1.6%)	
Other	242 (1.6%)	11 (1.6%)	
Missing	312 (2.1%)	22 (3.1%)	

**Table 3:** Mean and median waiting times to first assessment and treatment, stratified by age.

	Time to first assessment		*p* Value^a^	Time to first treatment		*P* value
	18–65	>65		18–65	>65	
Mean days (SD)	47.4 (54.32)	41.3 (46.90)	*p* < 0.01	52.1 (58)	44.3 (51.15)	*p* < 0.001
Median days (IQR)	29 (14–58)	27 (14–49)	*p* < 0.01	34 (16–65)	29 (14–57)	*p* < 0.001
STEP 2	28 (14–56)	25 (14–45)	*p* < 0.05	31 (16–62)	28 (15–52)	*p* < 0.01
STEP 3	32 (15–68)	30 (13–66)	*p* > 0.05	38 (18–76)	36 (13–70)	*p* < 0.05

a *p* Values were calculated using *t*-test for mean days, and Mann–Whitney *U* test for median values.

**Table 4:** *n* (%) of Recovery for depression and anxiety by age group and PCT.

		PHQ-9 recovered	*p* Value	GAD-7 recovered	*p* Value	Overall recovery	*p* Value
PCT A	18–65	914 (42.2)		1055 (48.9)		747 (34.6)	
	66–85	58 (50.0)	0.10	66 (56.9)	0.092	48 (41.4)	0.14
PCT B	18–65	648 (39.3)		687 (41.7)		471 (28.6)	
	66–85	34 (39.5)	0.96	39 (45.3)	0.50	60 (69.8)	0.74
PCT C	18–65	635 (32.3)		736 (37.5)		475 (24.2)	
	66–85	24 (37.5)	0.38	30 (46.9)	0.13	19 (29.7)	0.31
PCT D	18–65	520 (30.9)		581 (34.5)		402 (23.9)	
	66–85	39 (38.2)	0.12	53 (52.0)	**<0.001**	29 (28.43)	0.29
PCT E	18–65	1920 (41.0)		2141 (45.7)		1544 (32.9)	
	66–85	86 (40.8)	0.95	115 (54.5)	**0.012**	70 (33.2)	0.94
PCT F	18–65	955 (32.7)		1169 (40.0)		740 (25.3)	
	66–85	41 (32.5)	0.97	53 (42.0)	0.81	34 (27.0)	0.68
Overall	18–65	5592 (37.1)		6369 (42.28)		4379 (29.07)	
	66–85	282 (40.0)	0.12	356 (50.50)	**<0.001**	226 (32.06)	0.088

**Table 5:** Odds ratios of recovery adjusted for gender, age, primary care trust, max number of sessions and ethnicity.

		Recovery – depression		Recovery – anxiety		Overall recovery	
		OR^a^	OR^a^	OR^a^	OR^a^	OR^a^	OR^a^
Gender	*Female*	0.94 (0.89–1.00)	**0.91 (0.85–0.96)**	0.97 (0.92–1.02)	0.94 (0.89–1.00)	0.96 (0.90–1.02)	**0.91 (0.86–0.97)**
Age (baseline 18–65)	*66–85*	1.14 (0.97–1.33)	**1.30 (1.10–1.53)**	**1.42 (1.22–1.65)**	**1.42 (1.21–1.66)**	1.16 (0.98–1.37)	**1.31 (1.10–1.54)**
Max number of sessions		**1.06 (1.06–1.07)**		**1.06 (1.05–1.06)**		**1.06 (1.05–1.07)**	
Baseline Score^b^		**1.08 (1.07–1.08)**		**1.03 (1.02–1.04)**			
Treatment step^c^		**0.85 (0.79**–**0.92)**		**0.84 (0.78–0.90)**		**0.85 (0.79–0.92)**	
Dropout		**0.51 (0.47–0.56)**		**0.49 (0.44–0.53)**		**0.43 (0.39–0.48)**	

a Odds ratios have all been adjusted for primary care trust.

b Continuous variable, one point increase.

c dichotomous variable, reference: step 2.
