# Supplementary material for: Improving access to psychological therapies and older people: Findings from the Eastern Region
Source: Behav Res Ther. 2014 May;56(100):75–81. doi: 10.1016/j.brat.2014.03.008 (PMC4007011; doi:10.1016/j.brat.2014.03.008)
Supplement: Supplementary file 1 [file mmc1.docx]

|  | **Referral not accepted** | | | **GAD or PHQ missing** | | | **Only one assessment** | | | **Treated Sample** | | |
| --- | --- | --- | --- | --- | --- | --- | --- | --- | --- | --- | --- | --- |
| **Number of Individuals** | 7,142 |  | 9,787 | |  | 5,492 | |  | 16,236 | |  |  |
| **Number of sessions** | 16,105 |  | 52,860 | |  | 5,492 | |  | 99,402 | |  |  |
| **Median number of sessions/individual (IRQ Range)** | 1 | (1-2) | 2 | | (1-4) | 1 | | (1-1) | 5 | | (3-8) |  |
| **Mean session/individual (SD)** | 2.5 | (2.9) | 3.4 | | (4.4) | 1 | | (1) | 6.1 | | (4.7) |  |
| **Gender (Female %)** | 4,380 | (62.6%) | 6,133 | | (64.7%) | 3,533 | | (65.7%) | 10,747 | | (67.1%) |  |
| ***Missing*** | 123 | (1.7%) | 239 | | (2.4 %) | 90 | | (1.6 %) | 290 | | (1.8%) |  |
| **18-65** | 6,851 | (95.9%) | 9,483 | | (96.9 %) | 5,304 | | (96.6%) | 15,521 | | (95.7%) |  |
| **> 65** | 291 | (4.1%) | 304 | | (3.1 %) | 188 | | (3.4%) | 705 | | (4.3%) |  |
| **PCT A** | 544 | (7.6%) | 805 | | (8.2 %) | 475 | | (8.3%) | 2,275 | | (14.0%) |  |
| **B** | 444 | (6.2%) | 983 | | (10.4%) | 550 | | (10.0%) | 1,735 | | (10.7%) |  |
| **C** | 620 | (8.7%) | 780 | | (8.0 %) | 808 | | (14.7%) | 2,028 | | (12.5%) |  |
| **D** | 1,672 | (23.4%) | 1,334 | | (13.6 %) | 445 | | (8.1%) | 1,787 | | (11.0%) |  |
| **F** | 1,043 | (14.6%) | 3,136 | | (32.0 %) | 1,926 | | (35.1%) | 4,899 | | (30.2%) |  |
| **G** | 1,048 | (14.7%) | 1,046 | | (10.7 %) | 1,061 | | (19.3%) | 3,046 | | (18.8%) |  |

**Appendix 1 – Comparison of socio-demographics and number of sessions by different population samples**
